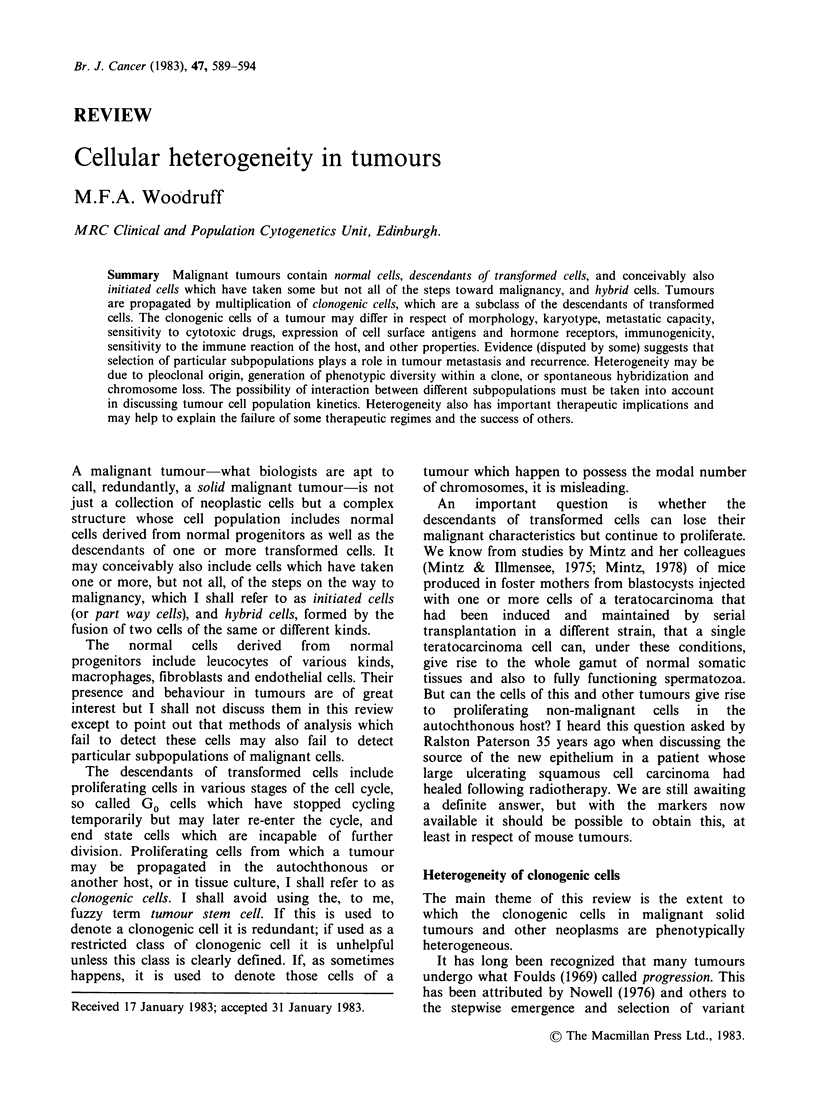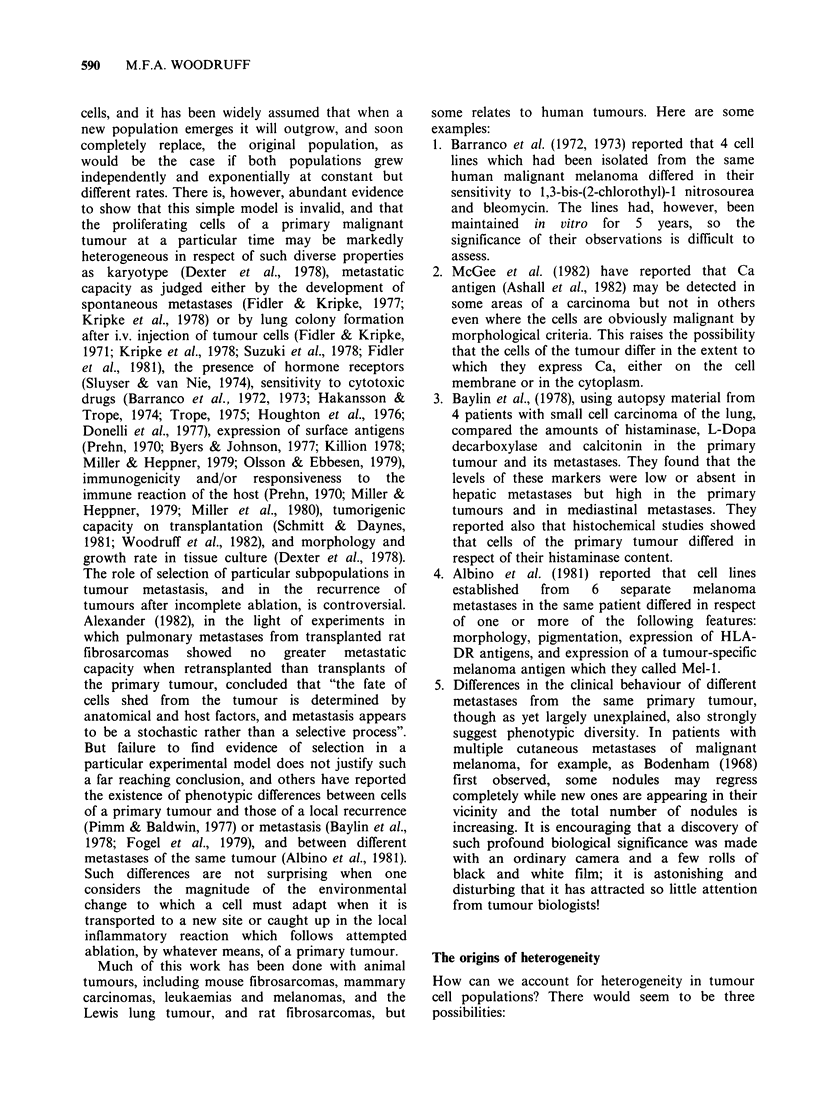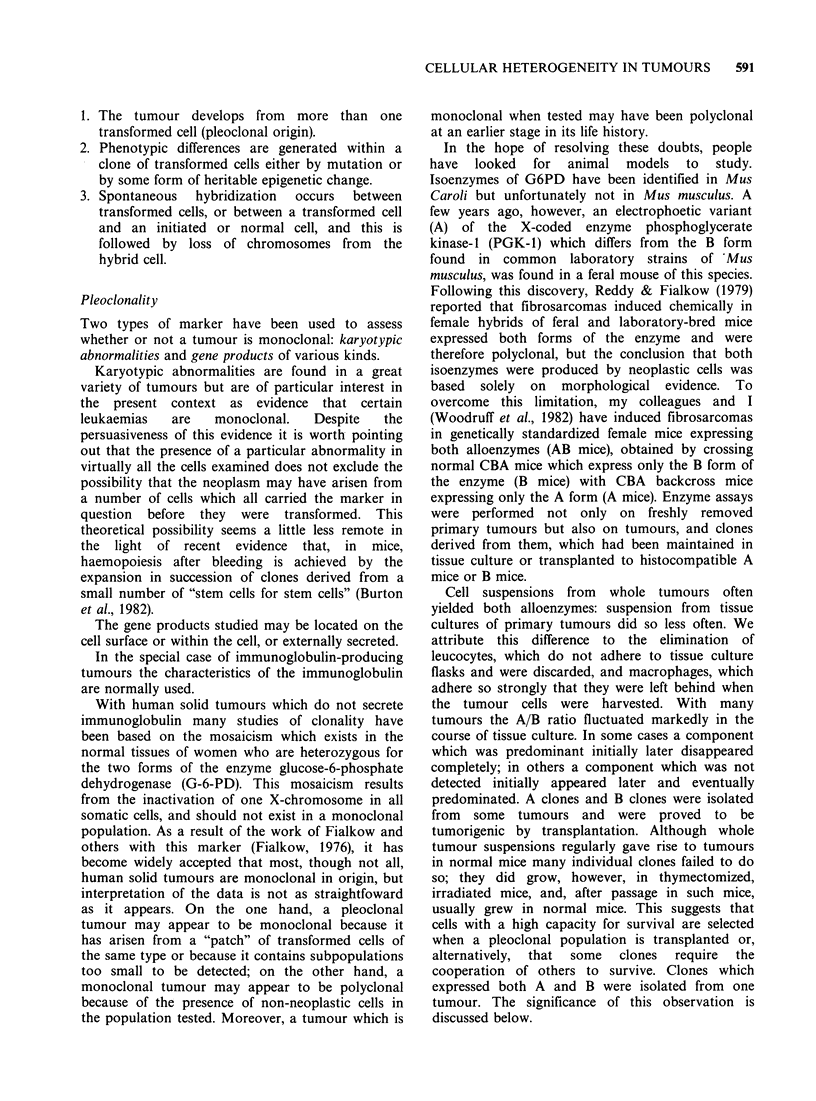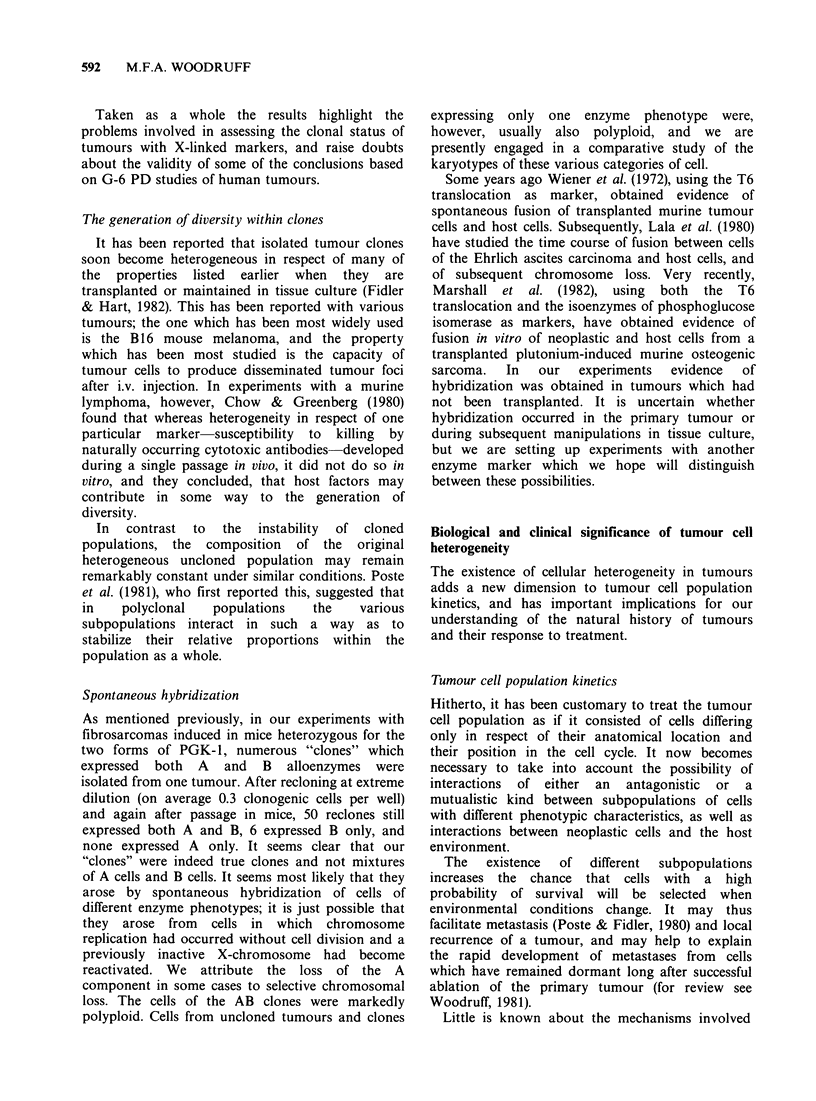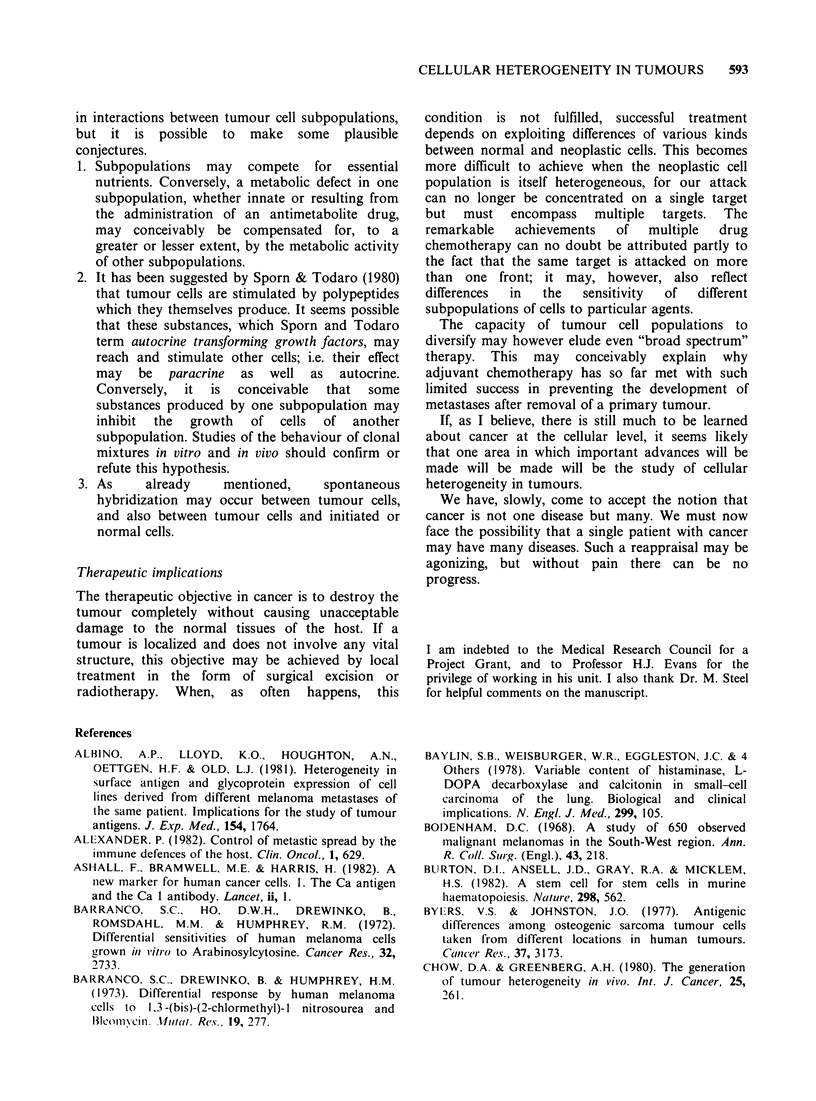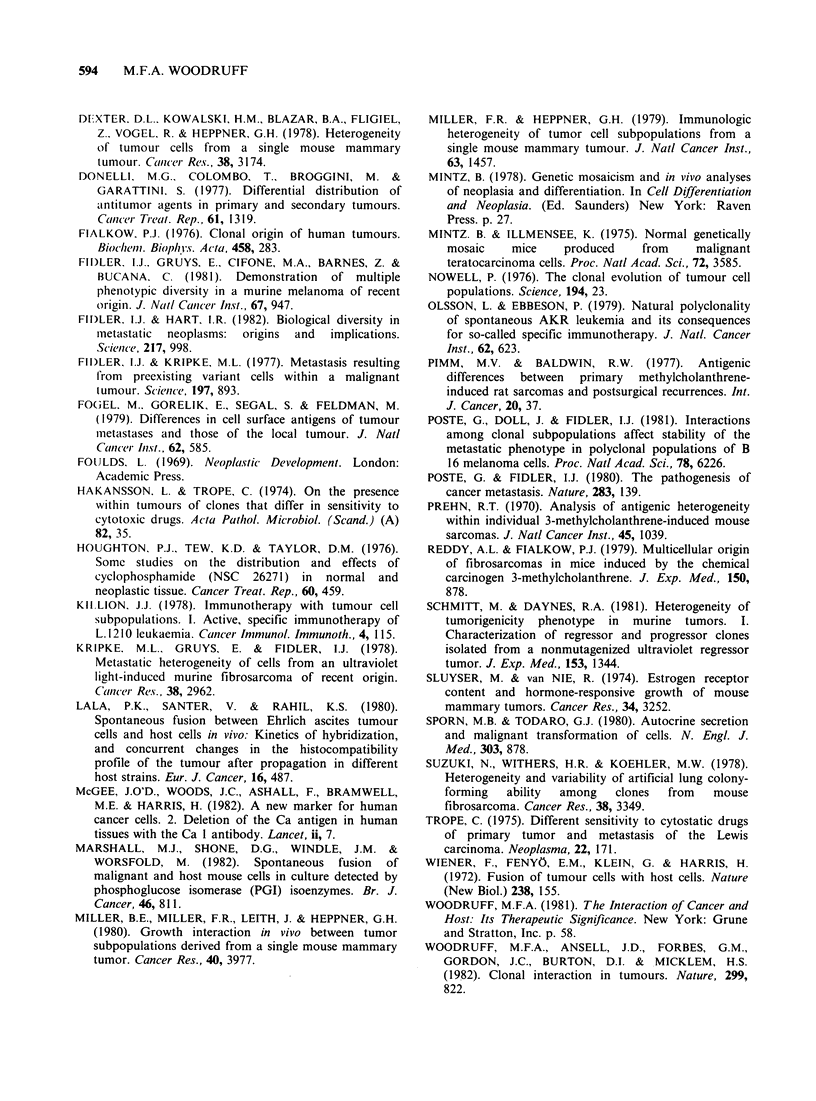# Cellular heterogeneity in tumours.

**DOI:** 10.1038/bjc.1983.96

**Published:** 1983-05

**Authors:** M. F. Woodruff

## Abstract

Malignant tumours contain normal cells, descendants of transformed cells, and conceivably also initiated cells which have taken some but not all of the steps toward malignancy, and hybrid cells. Tumours are propagated by multiplication of clonogenic cells, which are a subclass of the descendants of transformed cells. The clonogenic cells of a tumour may differ in respect of morphology, karyotype, metastatic capacity, sensitivity to cytotoxic drugs, expression of cell surface antigens and hormone receptors, immunogenicity, sensitivity to the immune reaction of the host, and other properties. Evidence (disputed by some) suggests that selection of particular subpopulations plays a role in tumour metastasis and recurrence. Heterogeneity may be due to pleoclonal origin, generation of phenotypic diversity within a clone, or spontaneous hybridization and chromosome loss. The possibility of interaction between different subpopulations must be taken into account in discussing tumour cell population kinetics. Heterogeneity also has important therapeutic implications and may help to explain the failure of some therapeutic regimes and the success of others.


Br. J. Cancer (1983), 47, 589–594

REVIEW

### Cellular heterogeneity in tumours

M.F.A. Woodruff

*MRC Clinical and Population Cytogenetics Unit, Edinburgh.*

**Summary** Malignant tumours contain *normal cells*, *descendants of transformed cells*, and conceivably also *initiated cells* which have taken some but not all of the steps toward malignancy, and *hybrid* cells. Tumours are propagated by multiplication of *clonogenic cells*, which are a subclass of the descendants of transformed cells. The clonogenic cells of a tumour may differ in respect of morphology, karyotype, metastatic capacity, sensitivity to cytotoxic drugs, expression of cell surface antigens and hormone receptors, immunogenicity, sensitivity to the immune reaction of the host, and other properties. Evidence (disputed by some) suggests that selection of particular subpopulations plays a role in tumour metastasis and recurrence. Heterogeneity may be due to pleoclonal origin, generation of phenotypic diversity within a clone, or spontaneous hybridization and chromosome loss. The possibility of interaction between different subpopulations must be taken into account in discussing tumour cell population kinetics. Heterogeneity also has important therapeutic implications and may help to explain the failure of some therapeutic regimes and the success of others.

A malignant tumour—what biologists are apt to call, redundantly, a *solid* malignant tumour—is not just a collection of neoplastic cells but a complex structure whose cell population includes normal cells derived from normal progenitors as well as the descendants of one or more transformed cells. It may conceivably also include cells which have taken one or more, but not all, of the steps on the way to malignancy, which I shall refer to as *initiated cells* (or *part way cells*), and *hybrid cells*, formed by the fusion of two cells of the same or different kinds.

The normal cells derived from normal progenitors include leucocytes of various kinds, macrophages, fibroblasts and endothelial cells. Their presence and behaviour in tumours are of great interest but I shall not discuss them in this review except to point out that methods of analysis which fail to detect these cells may also fail to detect particular subpopulations of malignant cells.

The descendants of transformed cells include proliferating cells in various stages of the cell cycle, so called $G_0$ cells which have stopped cycling temporarily but may later re-enter the cycle, and end state cells which are incapable of further division. Proliferating cells from which a tumour may be propagated in the autochthonous or another host, or in tissue culture, I shall refer to as *clonogenic cells*. I shall avoid using the, to me, fuzzy term *tumour stem cell*. If this is used to denote a clonogenic cell it is redundant; if used as a restricted class of clonogenic cell it is unhelpful unless this class is clearly defined. If, as sometimes happens, it is used to denote those cells of a tumour which happen to possess the modal number of chromosomes, it is misleading.

An important question is whether the descendants of transformed cells can lose their malignant characteristics but continue to proliferate. We know from studies by Mintz and her colleagues (Mintz & Illmensee, 1975; Mintz, 1978) of mice produced in foster mothers from blastocysts injected with one or more cells of a teratocarcinoma that had been induced and maintained by serial transplantation in a different strain, that a single teratocarcinoma cell can, under these conditions, give rise to the whole gamut of normal somatic tissues and also to fully functioning spermatozoa. But can the cells of this and other tumours give rise to proliferating non-malignant cells in the autochthonous host? I heard this question asked by Ralston Paterson 35 years ago when discussing the source of the new epithelium in a patient whose large ulcerating squamous cell carcinoma had healed following radiotherapy. We are still awaiting a definite answer, but with the markers now available it should be possible to obtain this, at least in respect of mouse tumours.

### Heterogeneity of clonogenic cells

The main theme of this review is the extent to which the clonogenic cells in malignant solid tumours and other neoplasms are phenotypically heterogeneous.

It has long been recognized that many tumours undergo what Foulds (1969) called *progression*. This has been attributed by Nowell (1976) and others to the stepwise emergence and selection of variant

Received 17 January 1983; accepted 31 January 1983.

© The Macmillan Press Ltd., 1983.

cells, and it has been widely assumed that when a new population emerges it will outgrow, and soon completely replace, the original population, as would be the case if both populations grew independently and exponentially at constant but different rates. There is, however, abundant evidence to show that this simple model is invalid, and that the proliferating cells of a primary malignant tumour at a particular time may be markedly heterogeneous in respect of such diverse properties as karyotype (Dexter *et al.*, 1978), metastatic capacity as judged either by the development of spontaneous metastases (Fidler & Kripke, 1977; Kripke *et al.*, 1978) or by lung colony formation after i.v. injection of tumour cells (Fidler & Kripke, 1971; Kripke *et al.*, 1978; Suzuki *et al.*, 1978; Fidler *et al.*, 1981), the presence of hormone receptors (Sluyser & van Nie, 1974), sensitivity to cytotoxic drugs (Barranco *et al.*, 1972, 1973; Hakansson & Trope, 1974; Trope, 1975; Houghton *et al.*, 1976; Donelli *et al.*, 1977), expression of surface antigens (Prehn, 1970; Byers & Johnson, 1977; Killion 1978; Miller & Heppner, 1979; Olsson & Ebbesen, 1979), immunogenicity and/or responsiveness to the immune reaction of the host (Prehn, 1970; Miller & Heppner, 1979; Miller *et al.*, 1980), tumorigenic capacity on transplantation (Schmitt & Daynes, 1981; Woodruff *et al.*, 1982), and morphology and growth rate in tissue culture (Dexter *et al.*, 1978). The role of selection of particular subpopulations in tumour metastasis, and in the recurrence of tumours after incomplete ablation, is controversial. Alexander (1982), in the light of experiments in which pulmonary metastases from transplanted rat fibrosarcomas showed no greater metastatic capacity when retransplanted than transplants of the primary tumour, concluded that "the fate of cells shed from the tumour is determined by anatomical and host factors, and metastasis appears to be a stochastic rather than a selective process". But failure to find evidence of selection in a particular experimental model does not justify such a far reaching conclusion, and others have reported the existence of phenotypic differences between cells of a primary tumour and those of a local recurrence (Pimm & Baldwin, 1977) or metastasis (Baylin *et al.*, 1978; Fogel *et al.*, 1979), and between different metastases of the same tumour (Albino *et al.*, 1981). Such differences are not surprising when one considers the magnitude of the environmental change to which a cell must adapt when it is transported to a new site or caught up in the local inflammatory reaction which follows attempted ablation, by whatever means, of a primary tumour.

Much of this work has been done with animal tumours, including mouse fibrosarcomas, mammary carcinomas, leukaemias and melanomas, and the Lewis lung tumour, and rat fibrosarcomas, but some relates to human tumours. Here are some examples:

1. Barranco *et al.* (1972, 1973) reported that 4 cell lines which had been isolated from the same human malignant melanoma differed in their sensitivity to 1,3-bis-(2-chlorothyl)-1 nitrosourea and bleomycin. The lines had, however, been maintained *in vitro* for 5 years, so the significance of their observations is difficult to assess.
2. McGee *et al.* (1982) have reported that Ca antigen (Ashall *et al.*, 1982) may be detected in some areas of a carcinoma but not in others even where the cells are obviously malignant by morphological criteria. This raises the possibility that the cells of the tumour differ in the extent to which they express Ca, either on the cell membrane or in the cytoplasm.
3. Baylin *et al.*, (1978), using autopsy material from 4 patients with small cell carcinoma of the lung, compared the amounts of histaminase, L-Dopa decarboxylase and calcitonin in the primary tumour and its metastases. They found that the levels of these markers were low or absent in hepatic metastases but high in the primary tumours and in mediastinal metastases. They reported also that histochemical studies showed that cells of the primary tumour differed in respect of their histaminase content.
4. Albino *et al.* (1981) reported that cell lines established from 6 separate melanoma metastases in the same patient differed in respect of one or more of the following features: morphology, pigmentation, expression of HLA-DR antigens, and expression of a tumour-specific melanoma antigen which they called Mel-1.
5. Differences in the clinical behaviour of different metastases from the same primary tumour, though as yet largely unexplained, also strongly suggest phenotypic diversity. In patients with multiple cutaneous metastases of malignant melanoma, for example, as Bodenham (1968) first observed, some nodules may regress completely while new ones are appearing in their vicinity and the total number of nodules is increasing. It is encouraging that a discovery of such profound biological significance was made with an ordinary camera and a few rolls of black and white film; it is astonishing and disturbing that it has attracted so little attention from tumour biologists!

### The origins of heterogeneity

How can we account for heterogeneity in tumour cell populations? There would seem to be three possibilities:

1. The tumour develops from more than one transformed cell (pleoclonal origin).
2. Phenotypic differences are generated within a clone of transformed cells either by mutation or by some form of heritable epigenetic change.
3. Spontaneous hybridization occurs between transformed cells, or between a transformed cell and an initiated or normal cell, and this is followed by loss of chromosomes from the hybrid cell.

*Pleoclonality*

Two types of marker have been used to assess whether or not a tumour is monoclonal: *karyotypic abnormalities* and *gene products* of various kinds.

Karyotypic abnormalities are found in a great variety of tumours but are of particular interest in the present context as evidence that certain leukaemias are monoclonal. Despite the persuasiveness of this evidence it is worth pointing out that the presence of a particular abnormality in virtually all the cells examined does not exclude the possibility that the neoplasm may have arisen from a number of cells which all carried the marker in question before they were transformed. This theoretical possibility seems a little less remote in the light of recent evidence that, in mice, haemopoiesis after bleeding is achieved by the expansion in succession of clones derived from a small number of "stem cells for stem cells" (Burton *et al*., 1982).

The gene products studied may be located on the cell surface or within the cell, or externally secreted.

In the special case of immunoglobulin-producing tumours the characteristics of the immunoglobulin are normally used.

With human solid tumours which do not secrete immunoglobulin many studies of clonality have been based on the mosaicism which exists in the normal tissues of women who are heterozygous for the two forms of the enzyme glucose-6-phosphate dehydrogenase (G-6-PD). This mosaicism results from the inactivation of one X-chromosome in all somatic cells, and should not exist in a monoclonal population. As a result of the work of Fialkow and others with this marker (Fialkow, 1976), it has become widely accepted that most, though not all, human solid tumours are monoclonal in origin, but interpretation of the data is not as straightfoward as it appears. On the one hand, a pleoclonal tumour may appear to be monoclonal because it has arisen from a "patch" of transformed cells of the same type or because it contains subpopulations too small to be detected; on the other hand, a monoclonal tumour may appear to be polyclonal because of the presence of non-neoplastic cells in the population tested. Moreover, a tumour which is monoclonal when tested may have been polyclonal at an earlier stage in its life history.

In the hope of resolving these doubts, people have looked for animal models to study. Isoenzymes of G6PD have been identified in *Mus Caroli* but unfortunately not in *Mus musculus*. A few years ago, however, an electrophoetic variant (A) of the X-coded enzyme phosphoglycerate kinase-1 (PGK-1) which differs from the B form found in common laboratory strains of *Mus musculus*, was found in a feral mouse of this species. Following this discovery, Reddy & Fialkow (1979) reported that fibrosarcomas induced chemically in female hybrids of feral and laboratory-bred mice expressed both forms of the enzyme and were therefore polyclonal, but the conclusion that both isoenzymes were produced by neoplastic cells was based solely on morphological evidence. To overcome this limitation, my colleagues and I (Woodruff *et al*., 1982) have induced fibrosarcomas in genetically standardized female mice expressing both alloenzymes (AB mice), obtained by crossing normal CBA mice which express only the B form of the enzyme (B mice) with CBA backcross mice expressing only the A form (A mice). Enzyme assays were performed not only on freshly removed primary tumours but also on tumours, and clones derived from them, which had been maintained in tissue culture or transplanted to histocompatible A mice or B mice.

Cell suspensions from whole tumours often yielded both alloenzymes: suspension from tissue cultures of primary tumours did so less often. We attribute this difference to the elimination of leucocytes, which do not adhere to tissue culture flasks and were discarded, and macrophages, which adhere so strongly that they were left behind when the tumour cells were harvested. With many tumours the A/B ratio fluctuated markedly in the course of tissue culture. In some cases a component which was predominant initially later disappeared completely; in others a component which was not detected initially appeared later and eventually predominated. A clones and B clones were isolated from some tumours and were proved to be tumorigenic by transplantation. Although whole tumour suspensions regularly gave rise to tumours in normal mice many individual clones failed to do so; they did grow, however, in thymectomized, irradiated mice, and, after passage in such mice, usually grew in normal mice. This suggests that cells with a high capacity for survival are selected when a pleoclonal population is transplanted or, alternatively, that some clones require the cooperation of others to survive. Clones which expressed both A and B were isolated from one tumour. The significance of this observation is discussed below.

Taken as a whole the results highlight the problems involved in assessing the clonal status of tumours with X-linked markers, and raise doubts about the validity of some of the conclusions based on G-6 PD studies of human tumours.

*The generation of diversity within clones*

It has been reported that isolated tumour clones soon become heterogeneous in respect of many of the properties listed earlier when they are transplanted or maintained in tissue culture (Fidler & Hart, 1982). This has been reported with various tumours; the one which has been most widely used is the B16 mouse melanoma, and the property which has been most studied is the capacity of tumour cells to produce disseminated tumour foci after i.v. injection. In experiments with a murine lymphoma, however, Chow & Greenberg (1980) found that whereas heterogeneity in respect of one particular marker—susceptibility to killing by naturally occurring cytotoxic antibodies—developed during a single passage *in vivo*, it did not do so *in vitro*, and they concluded, that host factors may contribute in some way to the generation of diversity.

In contrast to the instability of cloned populations, the composition of the original heterogeneous uncloned population may remain remarkably constant under similar conditions. Poste *et al.* (1981), who first reported this, suggested that in polyclonal populations the various subpopulations interact in such a way as to stabilize their relative proportions within the population as a whole.

*Spontaneous hybridization*

As mentioned previously, in our experiments with fibrosarcomas induced in mice heterozygous for the two forms of PGK-1, numerous "clones" which expressed both A and B alloenzymes were isolated from one tumour. After recloning at extreme dilution (on average 0.3 clonogenic cells per well) and again after passage in mice, 50 reclones still expressed both A and B, 6 expressed B only, and none expressed A only. It seems clear that our "clones" were indeed true clones and not mixtures of A cells and B cells. It seems most likely that they arose by spontaneous hybridization of cells of different enzyme phenotypes; it is just possible that they arose from cells in which chromosome replication had occurred without cell division and a previously inactive X-chromosome had become reactivated. We attribute the loss of the A component in some cases to selective chromosomal loss. The cells of the AB clones were markedly polyploid. Cells from uncloned tumours and clones expressing only one enzyme phenotype were, however, usually also polyploid, and we are presently engaged in a comparative study of the karyotypes of these various categories of cell.

Some years ago Wiener *et al.* (1972), using the T6 translocation as marker, obtained evidence of spontaneous fusion of transplanted murine tumour cells and host cells. Subsequently, Lala *et al.* (1980) have studied the time course of fusion between cells of the Ehrlich ascites carcinoma and host cells, and of subsequent chromosome loss. Very recently, Marshall *et al.* (1982), using both the T6 translocation and the isoenzymes of phosphoglucose isomerase as markers, have obtained evidence of fusion *in vitro* of neoplastic and host cells from a transplanted plutonium-induced murine osteogenic sarcoma. In our experiments evidence of hybridization was obtained in tumours which had not been transplanted. It is uncertain whether hybridization occurred in the primary tumour or during subsequent manipulations in tissue culture, but we are setting up experiments with another enzyme marker which we hope will distinguish between these possibilities.

**Biological and clinical significance of tumour cell heterogeneity**

The existence of cellular heterogeneity in tumours adds a new dimension to tumour cell population kinetics, and has important implications for our understanding of the natural history of tumours and their response to treatment.

*Tumour cell population kinetics*

Hitherto, it has been customary to treat the tumour cell population as if it consisted of cells differing only in respect of their anatomical location and their position in the cell cycle. It now becomes necessary to take into account the possibility of interactions of either an antagonistic or a mutualistic kind between subpopulations of cells with different phenotypic characteristics, as well as interactions between neoplastic cells and the host environment.

The existence of different subpopulations increases the chance that cells with a high probability of survival will be selected when environmental conditions change. It may thus facilitate metastasis (Poste & Fidler, 1980) and local recurrence of a tumour, and may help to explain the rapid development of metastases from cells which have remained dormant long after successful ablation of the primary tumour (for review see Woodruff, 1981).

Little is known about the mechanisms involved in interactions between tumour cell subpopulations, but it is possible to make some plausible conjectures.

1. Subpopulations may compete for essential nutrients. Conversely, a metabolic defect in one subpopulation, whether innate or resulting from the administration of an antimetabolite drug, may conceivably be compensated for, to a greater or lesser extent, by the metabolic activity of other subpopulations.
2. It has been suggested by Sporn & Todaro (1980) that tumour cells are stimulated by polypeptides which they themselves produce. It seems possible that these substances, which Sporn and Todaro term *autocrine transforming growth factors*, may reach and stimulate other cells; i.e. their effect may be *paracrine* as well as autocrine. Conversely, it is conceivable that some substances produced by one subpopulation may inhibit the growth of cells of another subpopulation. Studies of the behaviour of clonal mixtures *in vitro* and *in vivo* should confirm or refute this hypothesis.
3. As already mentioned, spontaneous hybridization may occur between tumour cells, and also between tumour cells and initiated or normal cells.

*Therapeutic implications*

The therapeutic objective in cancer is to destroy the tumour completely without causing unacceptable damage to the normal tissues of the host. If a tumour is localized and does not involve any vital structure, this objective may be achieved by local treatment in the form of surgical excision or radiotherapy. When, as often happens, this condition is not fulfilled, successful treatment depends on exploiting differences of various kinds between normal and neoplastic cells. This becomes more difficult to achieve when the neoplastic cell population is itself heterogeneous, for our attack can no longer be concentrated on a single target but must encompass multiple targets. The remarkable achievements of multiple drug chemotherapy can no doubt be attributed partly to the fact that the same target is attacked on more than one front; it may, however, also reflect differences in the sensitivity of different subpopulations of cells to particular agents.

The capacity of tumour cell populations to diversify may however elude even "broad spectrum" therapy. This may conceivably explain why adjuvant chemotherapy has so far met with such limited success in preventing the development of metastases after removal of a primary tumour.

If, as I believe, there is still much to be learned about cancer at the cellular level, it seems likely that one area in which important advances will be made will be made will be the study of cellular heterogeneity in tumours.

We have, slowly, come to accept the notion that cancer is not one disease but many. We must now face the possibility that a single patient with cancer may have many diseases. Such a reappraisal may be agonizing, but without pain there can be no progress.

I am indebted to the Medical Research Council for a Project Grant, and to Professor H.J. Evans for the privilege of working in his unit. I also thank Dr. M. Steel for helpful comments on the manuscript.

**References**

ALBINO, A.P., LLOYD, K.O., HOUGHTON, A.N., OETTGEN, H.F. & OLD, L.J. (1981). Heterogeneity in surface antigen and glycoprotein expression of cell lines derived from different melanoma metastases of the same patient. Implications for the study of tumour antigens. *J. Exp. Med.*, **154**, 1764.

ALEXANDER, P. (1982). Control of metastic spread by the immune defences of the host. *Clin. Oncol.*, **1**, 629.

ASHALL, F., BRAMWELL, M.E. & HARRIS, H. (1982). A new marker for human cancer cells. 1. The Ca antigen and the Ca 1 antibody. *Lancet*, **ii**, 1.

BARRANCO, S.C., HO, D.W.H., DREWINKO, B., ROMSDAHL, M.M. & HUMPHREY, R.M. (1972). Differential sensitivities of human melanoma cells grown *in vitro* to Arabinosylcytosine. *Cancer Res.*, **32**, 2733.

BARRANCO, S.C., DREWINKO, B. & HUMPHREY, H.M. (1973). Differential response by human melanoma cells to 1,3-(bis)-(2-chlormethyl)-1 nitrosourea and Bleomycin. *Mutat. Res.*, **19**, 277.

BAYLIN, S.B., WEISBURGER, W.R., EGGLESTON, J.C. & 4 Others (1978). Variable content of histaminase, L-DOPA decarboxylase and calcitonin in small-cell carcinoma of the lung. Biological and clinical implications. *N. Engl. J. Med.*, **299**, 105.

BODENHAM, D.C. (1968). A study of 650 observed malignant melanomas in the South-West region. *Ann. R. Coll. Surg.* (Engl.), **43**, 218.

BURTON, D.I., ANSELL, J.D., GRAY, R.A. & MICKLEM, H.S. (1982). A stem cell for stem cells in murine haematopoiesis. *Nature*, **298**, 562.

BYERS, V.S. & JOHNSTON, J.O. (1977). Antigenic differences among osteogenic sarcoma tumour cells taken from different locations in human tumours. *Cancer Res.*, **37**, 3173.

CHOW, D.A. & GREENBERG, A.H. (1980). The generation of tumour heterogeneity *in vivo*. *Int. J. Cancer*, **25**, 261.

DEXTER, D.L., KOWALSKI, H.M., BLAZAR, B.A., FLIGIEL, Z., VOGEL, R. & HEPPNER, G.H. (1978). Heterogeneity of tumour cells from a single mouse mammary tumour. *Cancer Res.*, **38**, 3174.

DONELLI, M.G., COLOMBO, T., BROGGINI, M. & GARATTINI, S. (1977). Differential distribution of antitumor agents in primary and secondary tumours. *Cancer Treat. Rep.*, **61**, 1319.

FIALKOW, P.J. (1976). Clonal origin of human tumours. *Biochem. Biophys. Acta*, **458**, 283.

FIDLER, I.J., GRUYS, E., CIFONE, M.A., BARNES, Z. & BUCANA, C. (1981). Demonstration of multiple phenotypic diversity in a murine melanoma of recent origin. *J. Natl Cancer Inst.*, **67**, 947.

FIDLER, I.J. & HART, I.R. (1982). Biological diversity in metastatic neoplasms: origins and implications. *Science*, **217**, 998.

FIDLER, I.J. & KRIPKE, M.L. (1977). Metastasis resulting from preexisting variant cells within a malignant tumour. *Science*, **197**, 893.

FOGEL, M., GORELIK, E., SEGAL, S. & FELDMAN, M. (1979). Differences in cell surface antigens of tumour metastases and those of the local tumour. *J. Natl Cancer Inst.*, **62**, 585.

FOULDS, L. (1969). *Neoplastic Development*. London: Academic Press.

HAKANSSON, L. & TROPE, C. (1974). On the presence within tumours of clones that differ in sensitivity to cytotoxic drugs. *Acta Pathol. Microbiol. (Scand.)* (A) **82**, 35.

HOUGHTON, P.J., TEW, K.D. & TAYLOR, D.M. (1976). Some studies on the distribution and effects of cyclophosphamide (NSC 26271) in normal and neoplastic tissue. *Cancer Treat. Rep.*, **60**, 459.

KILLION, J.J. (1978). Immunotherapy with tumour cell subpopulations. I. Active, specific immunotherapy of L.1210 leukaemia. *Cancer Immunol. Immunoth.*, **4**, 115.

KRIPKE, M.L., GRUYS, E. & FIDLER, I.J. (1978). Metastatic heterogeneity of cells from an ultraviolet light-induced murine fibrosarcoma of recent origin. *Cancer Res.*, **38**, 2962.

LALA, P.K., SANTER, V. & RAHIL, K.S. (1980). Spontaneous fusion between Ehrlich ascites tumour cells and host cells *in vivo*: Kinetics of hybridization, and concurrent changes in the histocompatibility profile of the tumour after propagation in different host strains. *Eur. J. Cancer*, **16**, 487.

McGEE, J.O'D., WOODS, J.C., ASHALL, F., BRAMWELL, M.E. & HARRIS, H. (1982). A new marker for human cancer cells. 2. Deletion of the Ca antigen in human tissues with the Ca 1 antibody. *Lancet*, **ii**, 7.

MARSHALL, M.J., SHONE, D.G., WINDLE, J.M. & WORSFOLD, M. (1982). Spontaneous fusion of malignant and host mouse cells in culture detected by phosphoglucose isomerase (PGI) isoenzymes. *Br. J. Cancer*, **46**, 811.

MILLER, B.E., MILLER, F.R., LEITH, J. & HEPPNER, G.H. (1980). Growth interaction *in vivo* between tumor subpopulations derived from a single mouse mammary tumor. *Cancer Res.*, **40**, 3977.

MILLER, F.R. & HEPPNER, G.H. (1979). Immunologic heterogeneity of tumor cell subpopulations from a single mouse mammary tumour. *J. Natl Cancer Inst.*, **63**, 1457.

MINTZ, B. (1978). Genetic mosaicism and *in vivo* analyses of neoplasia and differentiation. In *Cell Differentiation and Neoplasia*. (Ed. Saunders) New York: Raven Press. p. 27.

MINTZ, B. & ILLMENSEE, K. (1975). Normal genetically mosaic mice produced from malignant teratocarcinoma cells. *Proc. Natl Acad. Sci.*, **72**, 3585.

NOWELL, P. (1976). The clonal evolution of tumour cell populations. *Science*, **194**, 23.

OLSSON, L. & EBBESON, P. (1979). Natural polyclonality of spontaneous AKR leukemia and its consequences for so-called specific immunotherapy. *J. Natl. Cancer Inst.*, **62**, 623.

PIMM, M.V. & BALDWIN, R.W. (1977). Antigenic differences between primary methylcholanthrene-induced rat sarcomas and postsurgical recurrences. *Int. J. Cancer*, **20**, 37.

POSTE, G., DOLL, J. & FIDLER, I.J. (1981). Interactions among clonal subpopulations affect stability of the metastatic phenotype in polyclonal populations of B 16 melanoma cells. *Proc. Natl Acad. Sci.*, **78**, 6226.

POSTE, G. & FIDLER, I.J. (1980). The pathogenesis of cancer metastasis. *Nature*, **283**, 139.

PREHN, R.T. (1970). Analysis of antigenic heterogeneity within individual 3-methylcholanthrene-induced mouse sarcomas. *J. Natl Cancer Inst.*, **45**, 1039.

REDDY, A.L. & FIALKOW, P.J. (1979). Multicellular origin of fibrosarcomas in mice induced by the chemical carcinogen 3-methylcholanthrene. *J. Exp. Med.*, **150**, 878.

SCHMITT, M. & DAYNES, R.A. (1981). Heterogeneity of tumorigenicity phenotype in murine tumors. I. Characterization of regressor and progressor clones isolated from a nonmutagenized ultraviolet regressor tumor. *J. Exp. Med.*, **153**, 1344.

SLUYSER, M. & van NIE, R. (1974). Estrogen receptor content and hormone-responsive growth of mouse mammary tumors. *Cancer Res.*, **34**, 3252.

SPORN, M.B. & TODARO, G.J. (1980). Autocrine secretion and malignant transformation of cells. *N. Engl. J. Med.*, **303**, 878.

SUZUKI, N., WITHERS, H.R. & KOEHLER, M.W. (1978). Heterogeneity and variability of artificial lung colony-forming ability among clones from mouse fibrosarcoma. *Cancer Res.*, **38**, 3349.

TROPE, C. (1975). Different sensitivity to cytostatic drugs of primary tumor and metastasis of the Lewis carcinoma. *Neoplasma*, **22**, 171.

WIENER, F., FENYÖ, E.M., KLEIN, G. & HARRIS, H. (1972). Fusion of tumour cells with host cells. *Nature* (New Biol.) **238**, 155.

WOODRUFF, M.F.A. (1981). *The Interaction of Cancer and Host: Its Therapeutic Significance*. New York: Grune and Stratton, Inc. p. 58.

WOODRUFF, M.F.A., ANSELL, J.D., FORBES, G.M., GORDON, J.C., BURTON, D.I. & MICKLEM, H.S. (1982). Clonal interaction in tumours. *Nature*, **299**, 822.